# Chlorpromazine overcomes temozolomide resistance in glioblastoma by inhibiting Cx43 and essential DNA repair pathways

**DOI:** 10.1186/s12967-024-05501-3

**Published:** 2024-07-18

**Authors:** Paola Matarrese, Michele Signore, Barbara Ascione, Giulia Fanelli, Marco G. Paggi, Claudia Abbruzzese

**Affiliations:** 1 Center for Gender-Specific Medicine, Istituto Superiore di Sanità, 00161 Rome, Italy; 2 RPPA Unit, Proteomics Area, Core Facilities, Istituto Superiore di Sanità, 00161 Rome, Italy; 3 Cellular Networks and Molecular Therapeutic Targets, Proteomics Unit, IRCCS-Regina Elena National Cancer Institute, Via Elio Chianesi 53, 00144 Rome, Italy

**Keywords:** Glioblastoma, Chlorpromazine, Temozolomide, Connexin-43, DNA damage repair, Chemoresistance

## Abstract

**Background:**

In the fight against GBM, drug repurposing emerges as a viable and time-saving approach to explore new treatment options. Chlorpromazine, an old antipsychotic medication, has recently arisen as a promising candidate for repositioning in GBM therapy in addition to temozolomide, the first-line standard of care. We previously demonstrated the antitumor efficacy of chlorpromazine and its synergistic effects with temozolomide in suppressing GBM cell malignant features in vitro. This prompted us to accomplish a Phase II clinical trial to evaluate the efficacy and safety of adding chlorpromazine to temozolomide in GBM patients with unmethylated MGMT gene promoter. In this in vitro study, we investigate the potential role of chlorpromazine in overcoming temozolomide resistance.

**Methods:**

In our experimental set, we analyzed Connexin-43 expression at both the transcriptional and protein levels in control- and chlorpromazine-treated GBM cells. DNA damage and subsequent repair were assessed by immunofluorescence of γ-H2AX and Reverse-Phase Protein microArrays in chlorpromazine treated GBM cell lines. To elucidate the relationship between DNA repair systems and chemoresistance, we analyzed a signature of DNA repair genes in GBM cells after treatment with chlorpromazine, temozolomide and Connexin-43 downregulation.

**Results:**

Chlorpromazine treatment significantly downregulated connexin-43 expression in GBM cells, consequently compromising connexin-dependent cellular resilience, and ultimately contributing to cell death. In line with this, we observed concordant post-translational modifications of molecular determinants involved in DNA damage and repair pathways. Our evaluation of DNA repair genes revealed that temozolomide elicited an increase, while chlorpromazine, as well as connexin-43 silencing, a decrease in DNA repair gene expression in GBM cells.

**Conclusions:**

Chlorpromazine potentiates the cytotoxic effects of the alkylating agent temozolomide through a mechanism involving downregulation of Cx43 expression and disruption of the cell cycle arrest essential for DNA repair processes. This finding suggests that chlorpromazine may be a potential therapeutic strategy to overcome TMZ resistance in GBM cells by inhibiting their DNA repair mechanisms.

**Supplementary Information:**

The online version contains supplementary material available at 10.1186/s12967-024-05501-3.

## Background

Glioblastoma (GBM) is the most lethal brain tumor in adults and carries a grim prognosis with a median overall survival of less than 15 months. Indeed, despite the current standard therapeutic approach, which combines surgical resection (when possible) followed by radiotherapy plus concomitant and adjuvant chemotherapy with temozolomide (TMZ) [1], patients almost invariably succumb due to disease recurrences. Therefore, there is an urgent need for novel more effective therapeutic strategies toward GBM, with drug repurposing emerging as a promising avenue. Drug repurposing offers the potential for safer, faster and more cost-effective treatments for GBM patients, providing a glimmer of hope for this devastating disease [2].

TMZ, presently the first-line chemotherapeutic against GBM, is an alkylating agent that induces single- and double-strand DNA breaks, leading to cell cycle arrest at the G2/M boundary and ultimately apoptotic cell death [3, 4]. In an attempt to repair TMZ-induced alterations, GBM cells activate a variety of DNA repair mechanisms, including (i) base excision repair (BER); (ii) the enzyme methylguanine-DNA methyltransferase (MGMT); (iii) mismatch repair (MMR). An efficient DNA repair system and/or elevated MGMT protein levels characteristic as observed in GBM patients carrying a hypo- or unmethylated *MGMT* gene, confer resistance towards both radiotherapy and TMZ [5], allowing GBM cell to evade treatment, thus increasing the likelihood of clinical relapse [4, 6, 7], circumstances in which glioma stem cells (GSC) play a key role [8, 9].

Previous investigations shed light on the potential role of pro-oxidant agents to enhance TMZ efficacy by inducing excessive generation of reactive oxygen species (ROS) and oxidative stress, thus overcoming intracellular defense mechanisms. This generated interest in combining TMZ with pro-oxidant drugs as a strategy to overcome TMZ-induced chemoresistance in GBM cells [10, 11].

Connexins are a family of transmembrane proteins that form gap junctions in vertebrates, where play an important role in cell-to-cell communication, working as channels, and in cytosol-extracellular space, working as hemichannels, thus facilitating the exchange of small molecules, coordinating cellular activities and homeostasis [12, 13].

Connexin-43 [Cx43 or gap junction A1 (GJA1)] has been identified as the gap junction protein involved in tumor microtubes (TM) communication in astrocytomas, facilitating tumor progression, network communication and resilience to adverse events [14]. Several studies have shown the important function of Cx43 in malignant glioma growth control and migration. While several reports assign Cx43 an oncosuppressor feature, recent findings have revealed an opposite role: ongoing research demonstrates that Cx43 is highly expressed in GBM, especially in GSCs, conferring a survival advantage to tumor cells [15–19]. Previous studies highlighted the importance of Cx43 in ROS resistance in astrocytes, suggesting a pivotal role for these cells in controlling oxidative stress. Indeed, Cx43 knockdown leads to increased ROS-induced astrocytic death [20, 21]. Furthermore, Cx43 can confer chemotherapeutic resistance to GBM cells. Glioma cells overexpressing Cx43 exhibit higher resistance to TMZ, while Cx43 knockdown, in the same model system, sensitizes these cells to TMZ, implying a crucial role for this connexin in modulating chemoresistance in GBM. These findings suggest that combining Cx43 inhibitors with TMZ could offer a valid therapeutic approach for TMZ-resistant GBM patients [15, 16, 19, 22–24]. Finally, Cx43 overexpression in tumor cells promotes migration and invasion, and its expression level positively correlates with invasive capacity in GBM cell lines [25–27]. In sustaining this functional role, Cx43 localizes in invadopodia and contributes, through its interactome, to their formation and function in GBM cells [28].

Chlorpromazine (CPZ), an FDA- and EMA-approved antipsychotic medication, has been a mainstay of clinical practice in psychiatric disorders for over seven decades, due to its well-characterized ability to antagonize dopamine at the level of the CNS dopamine receptor D2 (DRD2). In recent years, CPZ has also emerged as a promising antitumor agent, demonstrating efficacy against various in vitro-growing cancers, including GBM, where the drug is able to: (1) inhibit cell growth and proliferation; (2) induce nuclear aberrations; (3) create endoplasmic reticulum (ER) stress and ROS generation; (4) induce cytotoxic autophagy; (5) reduce stemness features; (6) interfere with GBM energy metabolism; (7) induce cancer cell death [29–33].

Based on the discovery of stimulatory synaptic connections between neurons and GBM mediated by monoamines and their receptors [34, 35], and on our previous work demonstrating the direct antitumor potential of CPZ and its synergistic effects with TMZ in restraining GBM growth in vitro [36], we carried out a Phase II multicenter clinical trial to evaluate the efficacy of CPZ in combination with TMZ in the first-line treatment of GBM patients with an unmethylated *MGMT* gene promoter, a group known to exhibit higher resistance to TMZ [37]. The present study delves into a novel mechanism underlying CPZ's anti-neoplastic properties, focusing on its potential role in circumventing TMZ resistance by hindering MGMT-independent DNA repair pathways.

## Materials and methods

### Cell lines

Anchorage-dependent GBM cell lines U-87 MG and U-251 MG, as well as hTERT-immortalized human retinal pigment epithelial cells hTERT RPE-1 (here described only as RPE-1), were cultured as previously reported [31]. Anchorage-independent TS#1 and TS#163 are patient-derived cell lines, previously characterized and cultured as described [38].

All cell lines, when treated, were exposed to a drug dose corresponding to their IC30 (Additional file 1).

### Drugs

CPZ was purchased, as “Largactil”, from Teofarma S.R.L., Valle Salimbene (PV), Italy, as a 25 mg/ml solution (78 mM). TMZ was purchased from Selleckchem (Houston, TX, USA) and diluted in DMSO as a 150 mM solution.

### Transfection of cell lines (Cx43 silencing)

Anchorage-dependent U-87 MG and RPE-1 cells were plated and transfected with 10 nM siRNA-Cx43 or negative control siRNA using Lipofectamine RNAiMAX (Invitrogen Thermo Fisher Scientific) following manufacturer’s instructions. On the other hand, TS#163 neurospheres were seeded in Stem Medium containing 2% Matrigel (Corning Matrigel Growth Factor Reduced Basement Membrane Matrix, Merck, Darmstadt, Germany) and then transfected for Cx43 silencing. siRNA reagents (Cx43 Silencer Select Validated siRNA and Silencer Select Negative Control#1 siRNA) were from Ambion (Austin, TX, USA). After 48 h silencing, cells were collected and used for RNA and protein determinations (Supplementary figure S1).

### RNA extraction and RT-PCR

All GBM cells and non-cancer RPE-1 cells, untreated or treated with CPZ and/or TMZ, as well as Cx43 silenced cells, were subjected to RNA extraction using miRNeasy Extraction Kit (QIAGEN, Hilden, Germany) successively employed for real-time polymerase chain reaction (RT-PCR) analyses to determine transcriptional expression of several genes. All RT-PCR data were quantified using the 2^−ΔΔCT^ method and values represent fold changes related to control cells, arbitrarily reported as 1.0. When NRF2 gene and ARE pathway genes were analyzed, CT values were normalized to ribosomal protein S18 (RPS18), while GAPDH was employed to normalize CT values of Cx43 and DNA repair genes. All primers used are listed in Additional file 2.

### Immunoblot analysis

Cells were lysed in RIPA buffer in the presence of protease and phosphatase inhibitors, then processed (SDS-PAGE + western blot) and the solid support was probed with the following reagents: anti-NRF2 rabbit monoclonal antibody (Abcam, 1:1000); anti-Cx43 rabbit polyclonal antibody (Sigma-Aldrich, 1:8000); anti-β-actin mouse monoclonal antibody (MP Biomedicals, 1:10,000); anti-GAPDH rabbit monoclonal antibody (Cell Signaling Technology, 1:1000).

### Immunofluorescence analysis

Cells were fixed with 4% paraformaldehyde and permeabilized by 0.5% (v/v) Triton X-100. To detect DNA damage, cells were incubated for 1 h at 4°C with a specific mouse monoclonal antibody (Novus Biologicals, LLC, CO, USA, 1:200) to γ-H2AX, a biomarker for DNA double-strand breaks. AlexaFluor 594-conjugated anti-mouse IgG was used as a secondary antibody (45 min incubation at RT). After washing, samples were counterstained with Hoechst 33,258 and then mounted in fluorescence mounting medium (Dako, Glostrup, Denmark). Images were acquired with intensified video microscopy (IVM) using an Olympus fluorescence microscope (Olympus Corporation, Milan, Italy) equipped with CoolLed pE-300-W (CoolLED Ltd., Andover, UK).

### Quantitative flow cytometry

*ROS.* ROS production was measured in living cells by the fluorogenic probe 2′,7′-dichlorodihydrofluorescein diacetate (DCFH_2_-DA, Molecular Probes, Invitrogen). Control and treated cells were stained with 5 µM CM-H_2_DCFDA in PBS, according to the manufacturer’s instructions, and incubated at 37°C for 30 min before acquisition on a cytometer. As a negative control, unstained cells were used.

*GSH.* The intracellular glutathione level (GSH) was detected by staining living cells with monochlorobimane (MCB, Molecular Probes), as previously described [39]. MCB was added to the cell suspension to a final concentration of 40 μM and the cells were maintained at room temperature in the dark for 20 min prior to analysis. As a negative control, unstained cells were used.

*Cx43 detection.* After washing in PBS, control and treated cells, fixed and permeabilized as above, were incubated for 1 h at 4°C with a specific primary rabbit polyclonal antibody (Sigma-Aldrich, 1:200). Cy5-conjugated anti-rabbit (Abcam) was used as secondary antibody for 45 min at RT. As negative control, we used cells incubated with total rabbit serum, followed by a Cy5-conjugated anti-rabbit.

*DNA damage detection*. After washing in PBS, control and treated cells were fixed and permeabilized as mentioned above, and then incubated for 1 h at 4°C with a specific primary mouse monoclonal anti-γ-H2AX antibody (Novus Biologicals, 1:200). Cy5-conjugated anti-mouse (Abcam) was used as a secondary antibody for 45 min at RT. As negative control, cells incubated with IgG isotype, followed by a Cy5-conjugated anti-mouse antibody were used.

Samples were acquired with a FACSCalibur cytometer (BD Biosciences Inc., San Diego, CA, USA) equipped with a 488 nm Argon laser and with a 635 nm red diode laser and analyzed using CellQuest software (BD Biosciences). For GSH quantification, samples were acquired with a LRS II cytometer (Becton & Dickinson, San Jose, CA, USA) equipped with a 488 nm Argon laser and a UVB laser and analyzed with DIVA software (Becton & Dickinson). At least 10,000 events for each sample were acquired. Data were analyzed using the Cell Quest Pro software (BD Biosciences) or the DIVA software (Becton Dickinson).

### Glutathione assay

Intracellular glutathione (GSH) and oxidized forms [oxidized glutathione (GSSG)] were measured in untreated and treated cells with the Glutathione Assay Kit (Cayman Chemical, Florence, Italy) following the manufacturer’s instructions, after deproteinization of the samples with metaphosphoric acid. For GSSG quantification, an aliquot of deproteinized samples was first incubated with 2-vinylpyridine to derivatize GSH. Reduced GSH levels were obtained by differences between total GSH and GSSG.

### RPPA analysis

Reverse-Phase Protein microArrays (RPPA) analysis was performed following established procedures [40, 41]. Briefly, protein extracts were resuspended in Laemmli sample buffer [42] at a final concentration of 0.5 mg/mL with 2.5% TCEP reducing agent (Thermo-Fisher Scientific) and boiled for 3’ prior to printing with an Aushon 2470 (Quanterix) microarrayer. RPPA samples were printed in technical triplicates onto nitrocellulose-coated slides (Grace Bio-labs). Total protein content of printed slides was measured using Sypro Ruby (ThermoFisher Scientific). Immunostaining was performed by means of an automated system (DAKO AutostainerLink 48) using selected, pre-validated antibodies and a commercially available signal amplifiction kit (Agilent/DAKO GenPoint). The tertiary reagent used for signal detection was streptavidin-conjugated IRDye680LT (LI-COR Biosciences). Stained slides were scanned by a Power Scanner (TECAN) and 16-bit images were analyzed via MicroVigene v5.2 software (VigeneTech) to detect spots and normalize signal. Graphical representation of RPPA data was performed by means of ‘R’ v4.1.2 (https://www.r-project.org/) (R Foundation for Statistical Computing) and ‘RStudio’ v2023.06 https://www.rstudio.com/ (Rstudio).

### Statistical analysis

All results obtained by Western blotting and Real-Time PCR were quantified as means ± Standard Deviation (SD) vs each control and statistical significance was performed with Student's t-test using GraphPad Prism v9 (GraphPad, San Diego, CA, USA).

The amount of ROS, GSH, Cx43, and γ-H2AX, evaluated by FACS analyses, was expressed as the median fluorescence intensity and data are reported as means ± Standard Deviation (SD). Collected data analysis was carried out with ANOVA one-way testing using GraphPad Prism v9.

All data were verified in at least three independent experiments. A p value less than 0.05 was considered as statistically significant; (*) p ≤ 0.05, (**) p ≤ 0.01 and (***) p ≤ 0.001.

## Results

### Pro-oxidant effect of CPZ and cellular antioxidant activity

CPZ exerts pro-oxidative effects in GBM cells by increasing ROS generation, inducing ER stress and triggering unfolded protein response (UPR), as demonstrated in our previous work [31].

#### A. CPZ induces free radicals production in GBM cells

**Fig. 1:**
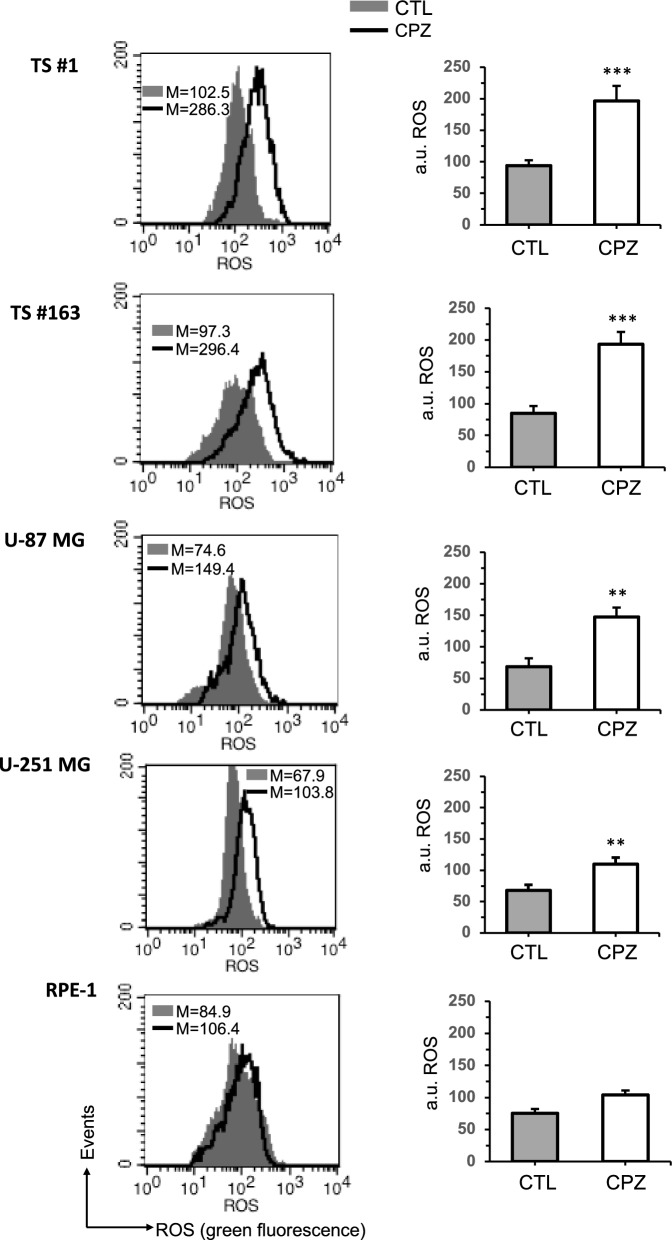
CPZ induces ROS production in GBM cells. All GBM cells and non tumoral RPE-1 cells were treated with solvent or CPZ for 48h and then subjected to FACS analyses. Left panels. Cytofluorimetric histograms of total ROS production obtained in a representative experiment. Right panels. Bar graph showing ROS production obtained by pooling together measures acquired in 3 different experiments and reported as mean ± SD of the median fluorescence intensity. Asterisks denote statistical significance (*p < 0.05; **p < 0.01; ***p < 0.001)

Here we present additional results regarding the increase in free radicals production in GBM following CPZ exposure. Data were obtained by using DCFH_2_-DA, the most widely used fluorogenic probe for the semiquantitative detection of general oxidative stress [43]. Quantitative flow cytometry analyses (Fig. 1) revealed a significant increase in ROS production induced by CPZ in the anchorage-dependent cell lines U-87 MG and U-251 MG, as well as in TS#1 and TS#163 neurospheres. Conversely, in the non-cancerous RPE-1 cell line, no significant increase following CPZ exposure was observed.

These results prompted us to evaluate the antioxidant capacity in GBM cells and in RPE-1 non-cancer cells to control and restore redox homeostasis after CPZ-induced alterations. The effect of CPZ was thus evaluated considering several parameters related to the redox equilibrium.

#### B. CPZ induces NRF2 upregulation in GBM cells

The nuclear factor erythroid 2-related factor 2 (NRF2) plays a key role in protecting cells from oxidative stress and regulates antioxidant defense systems [44, 45] in a context where the interplay between ROS and NRF2 signaling pathways is implicated in carcinogenesis [45, 46]. NRF2 is under the transcriptional control of p62 and, in basal conditions, the level of cellular NRF2 protein remains constantly regulated by the ubiquitin proteasome system, while stress conditions activate the NRF2 pathway [47].

**Fig. 2:**
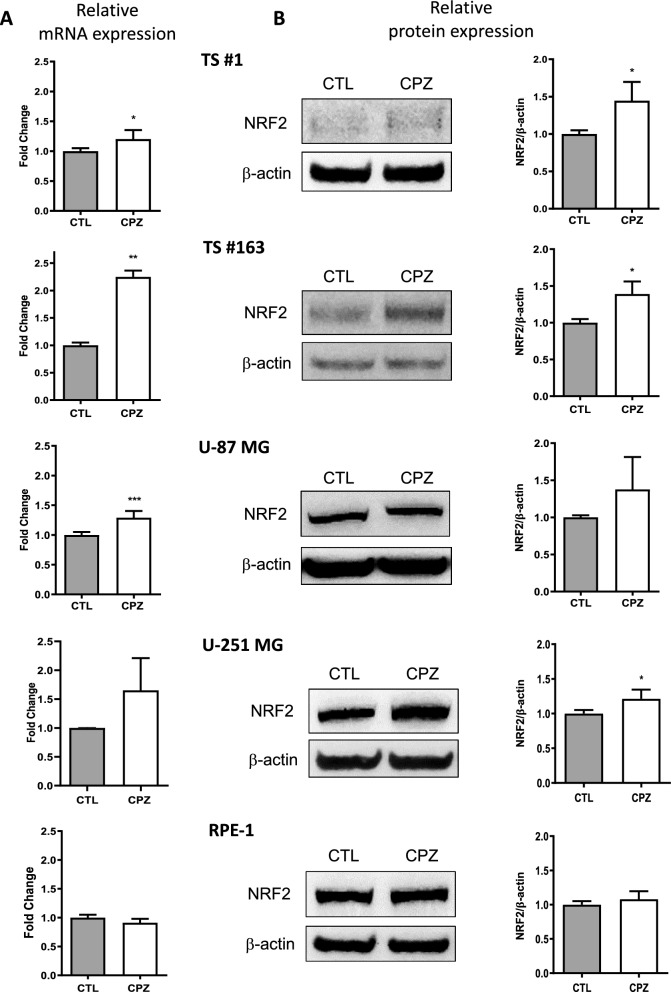
CPZ induces an increase of NRF2. **A** Relative mRNA expression of Nrf2 has been evaluated by means of qRT-PCR in untreated and CPZ-treated (for 24h) GBM cells and in non-cancer RPE-1 cell line. **B** Expression level of the protein NRF2, determined by Western blotting, in the same cells showing similar results. Histograms on the right quantify CPZ-induced protein increase, shown as mean ± SD, assessed by three independent experiments. Asterisks denote statistical significance (*p < 0.05; **p < 0.01; ***p < 0.001)

Both anchorage-dependent GBM cells and neurospheres exhibited a constant increase in NRF2 expression at both the transcriptional and protein levels upon CPZ treatment (Fig. 2A and B, respectively); in contrast, under the same conditions, RPE-1 non-cancer cells showed no significant modulation in NRF2 mRNA or protein expression.

#### C. CPZ induces upregulation of glutathione

**Fig. 3:**
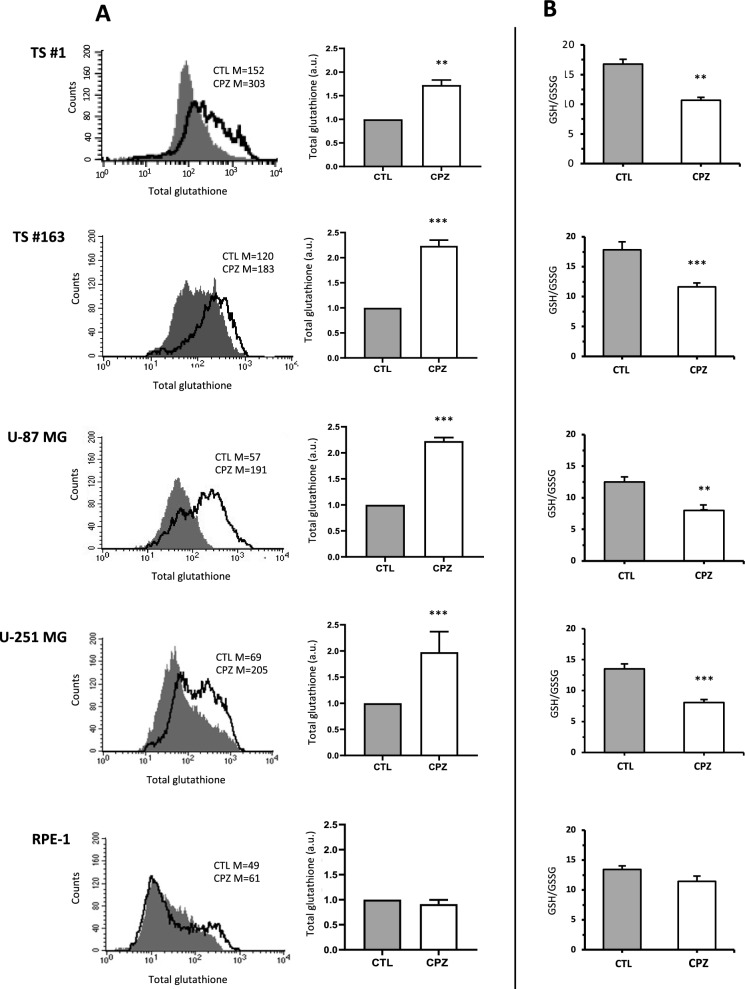
CPZ induces an upregulation of glutathione. **A** FACS analyses showed a significant increase of total glutathione amount in 48h CPZ-treated cells, when compared to controls, suggestive of an antioxidant effect, while the same impact was not apparent on the RPE-1 non-cancer cell line. **B** GSH/GSSG ratio, indicative of the oxidative balance, was determined via cytofluorimetric assay and relative expression levels represented as histograms. A significant decrease of GSH/GSSG ratio is evident only in GBM cells after CPZ treatment. Statistical significance is referred toward the Control (**p < 0.01; ***p < 0.001)

To evaluate the effect of CPZ on the antioxidant capacity of GBM cells, we assessed the amount of glutathione in all GBM cells with or without exposure to CPZ. As shown in the graphs and histograms in Fig. 3A, all GBM cells exhibited a significant increase in total glutathione content following CPZ-treatment, when compared with controls, an increase not observed in the RPE-1 non-cancer cell line. Notably, CPZ induced a significant associated decrease in the GSH/GSSG ratio, indicative of a state of oxidative stress, in GBM cells only (Fig. 3B).

#### D. CPZ activates the ARE pathway

**Fig. 4:**
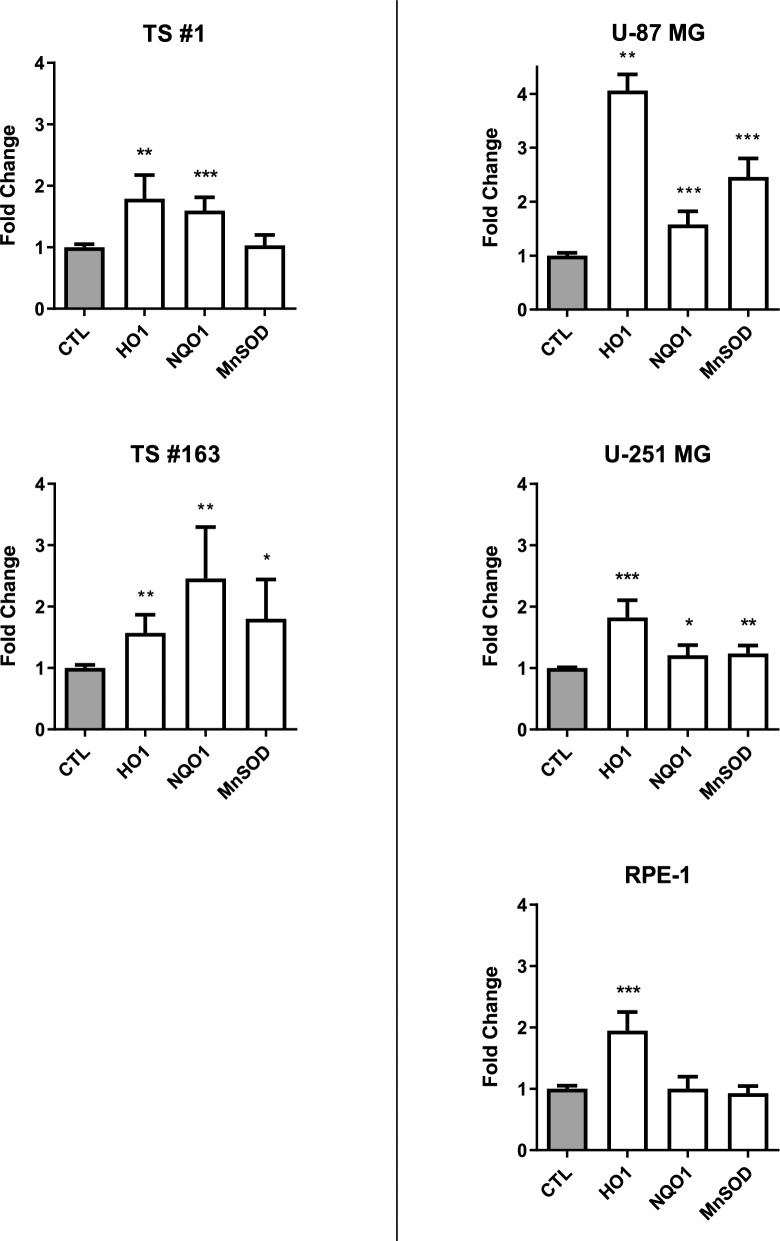
CPZ activates ARE pathway in GBM cells. Expression of genes involved in ARE pathway in the two GBM neurospheres (left of the panel) and in three anchorage-dependent cell lines (right of the panel). In all cases, determinations were performed via qRT-PCR after 24 h of exposure to CPZ. The histogram bars related to the control (CTL) values, normalized to 1.0 (gray columns), represent the fold-change increase for CPZ-treated cells. Data are presented as mean ± SD, along with statistical significance (*p < 0.05; **p < 0.01; ***p < 0.001)

As a sensor of oxidative stress, NRF2 signaling induces the antioxidant response element (ARE) pathway to activate defense factors and detoxify the cell. During the NRF2-ARE system activation, NRF2 dissociates from the repressor protein Keap1 and translocates into the nucleus to promote the transcription of the ARE-regulated genes, i.e., hemoxygenase 1 (HO-1), NAD(P)H dehydrogenase quinone 1 (NQO1) and manganese superoxide dismutase (MnSOD) [48, 49]. To assess the ability of CPZ to induce the ARE pathway, we treated anchorage-dependent GBM cells and neurospheres with a CPZ dose corresponding to their IC30; after 24 h of CPZ or solvent treatment, we extracted RNA and performed RT-PCR to examine the expression of ARE pathway-regulated genes. As shown in Fig. 4, CPZ induced significant upregulation of *HO1*, *NQO1* and *MnSOD*, all genes target of the ARE pathway and encoding cytoprotective, antioxidant and detoxifying enzymes. Under the same experimental conditions, the RPE-1 cell line showed a significant increase in only the HO-1 gene expression, suggesting that these cells might elicit a different cytoprotective mechanism in response to CPZ-induced oxidative stress and that might be more capable of recovering the damage.

### CPZ reduces Cx43 expression

**Fig. 5:**
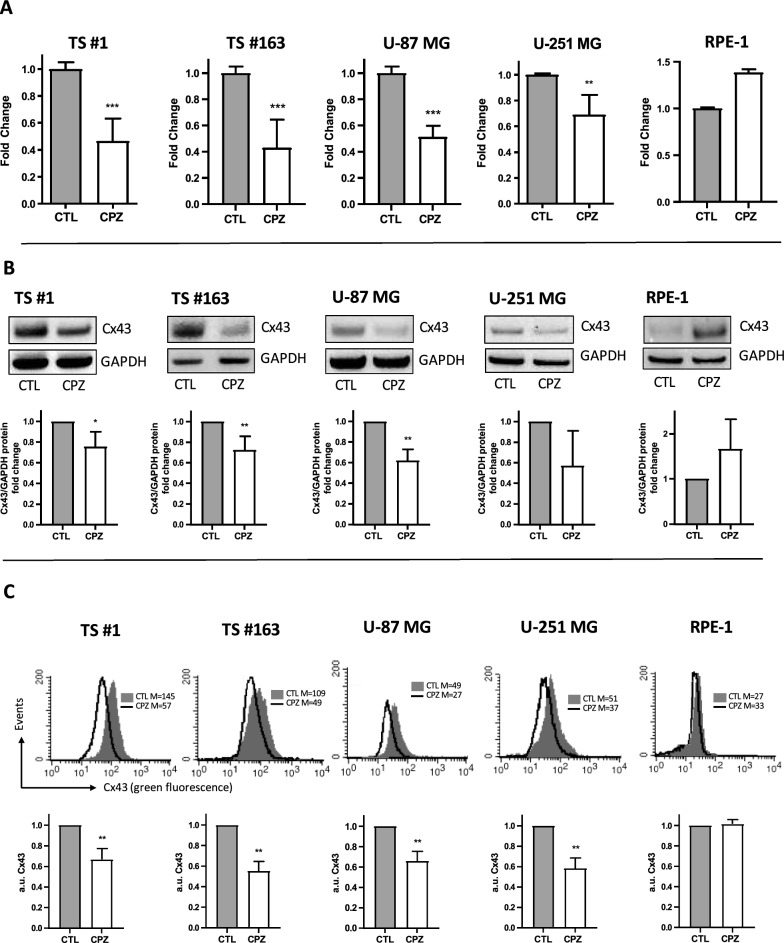
CPZ reduces Cx43 expression in GBM cells. Anchorage-dependent U-87 MG and U-251 MG GBM cells and RPE-1 non-cancer cells, as well as GBM-patients derived neurospheres, were exposed to CPZ or solvent (CTL) for 24h. Afterwards, CX43 expression was analyzed both at transcriptional level, by means RT-PCR (**A**), and at protein level, by means western blotting (**B**) and cytofluorimetric analyses (**C**). In all GBM cells we highlighted a relevant transcript and protein decrease of Cx43 expression, not appreciable in non-cancer cells. Statistical significance is indicated with asterisks (*p < 0.05; **p < 0.01; ***p < 0.001)

Cx43 is a gap junction protein involved in cellular responses to oxidative stress and TMZ resistance in glioma cells [15, 20]. As previously reported [16], when we treated all cell lines with TMZ, a significant increase in Cx43 expression was apparent (Supplementary figure S2). Conversely, as described above, exposure to CPZ induced a pro-oxidative status, triggering a cellular response involving defense and detoxifying mechanisms. Given the well-established neuroprotective role of Cx43 [20, 21], we evaluated its expression following exposure to CPZ. We treated anchorage-dependent GBM cells and neurospheres with their respective IC30 doses for 24 h and analyzed Cx43 expression at both the transcriptional level, by RT-PCR, and the protein level, by western blot and cytofluorimetric analysis. In all cases, we observed a significant decrease in Cx43 expression in GBM cells following CPZ exposure. Notably, under the same conditions, RPE-1 non-cancer cells didn’t show any reduction in Cx43 transcripts or protein levels (Fig. 5A–C).

Considering the crucial role of Cx43 in TMZ resistance [15, 50], these results prompted us to investigate the potential role of CPZ in overcoming TMZ resistance. In addition, in our previous work, we demonstrated a synergistic effect of CPZ with TMZ in restraining GBM growth [36].

### CPZ causes DNA damage and modulates DNA damage response

Since DNA damage response (DDR) may contribute to the hypersensitivity or resistance of cancer cells to genotoxic agents [51], and CPZ shows a synergy with TMZ [36], we investigated the effects of CPZ on DNA repair pathways.

#### A. CPZ increases DNA damage in TMZ-treated GBM cells

**Fig. 6:**
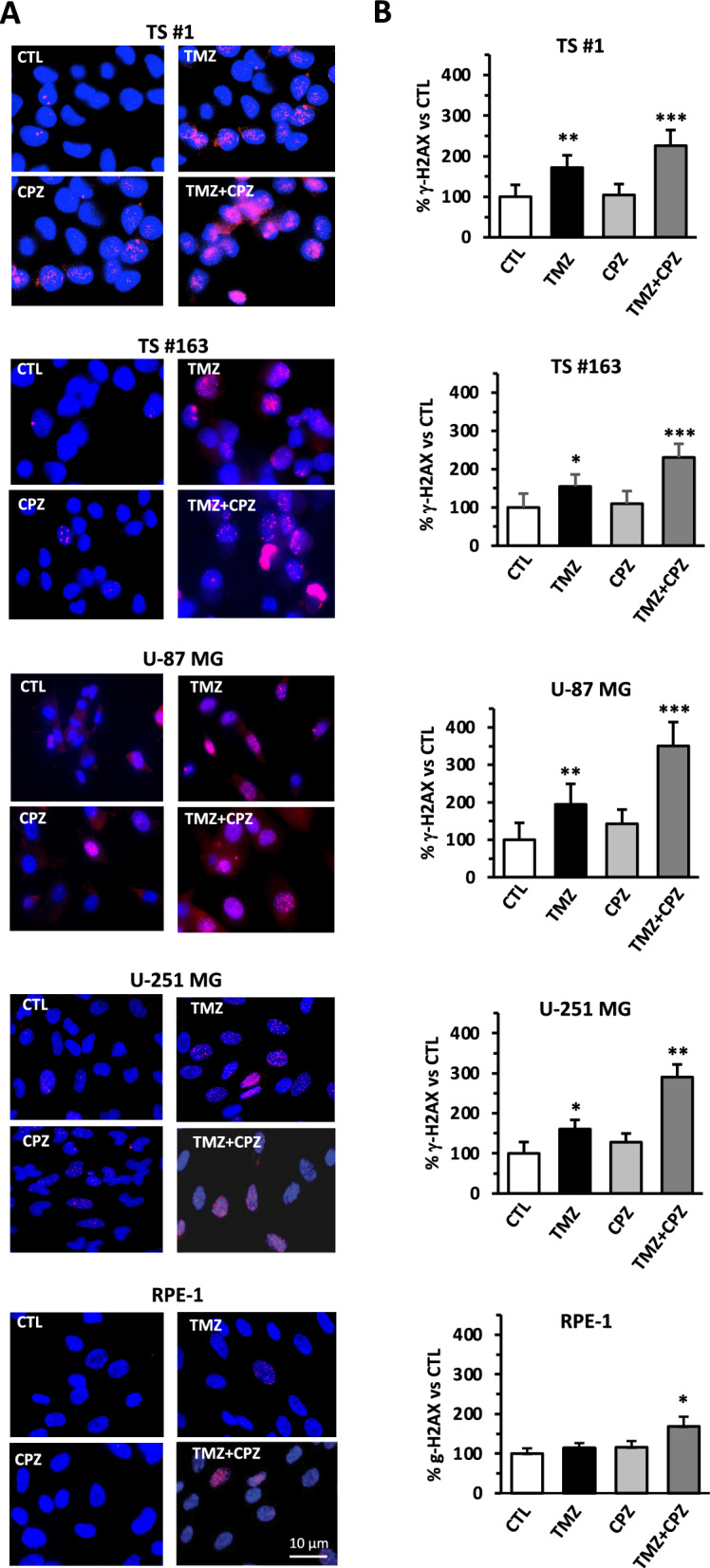
CPZ increases DNA damage in TMZ-treated GBM cells. Anchorage-dependent GBM cells, neurospheres and RPE-1 non-cancer cells were treated with CPZ or TMZ for 48h and 6 days respectively; when their combination was evaluated, cells were initially treated with an IC_30_ dose of TMZ for 96 h, then CPZ (IC_30_) was added for further 48 h. After that DNA damage was evaluated analyzing DSBs by way of histone H2AX analysis. **A** In representative immunofluorescence images, γ-H2AX foci are represented in red, while cell nuclei are represented in blue. In GBM cells, TMZ treatment induced a considerable increase of γ-H2AX adducts, further boosted by the TMZ + CPZ combination, while this outcome resulted less prominent in RPE-1 cells. **B** γ-H2AX foci analysis was also performed by means of cytofluorimetric examination. Histogram bars represent the adducts amount counted in at least 100 cells for each experimental point (magnification 500 ×). Data from three experiments with statistical significance are shown (*p < 0.05; **p < 0.01; ***p < 0.001)

Neurospheres, anchorage-dependent GBM cells and RPE-1 non-cancer cells were treated with CPZ, TMZ or their combination, then the presence of γ-H2AX foci, a readout of DSBs and thus DNA damage, was assessed using immunofluorescence and cytofluorimetric analysis. Immunofluorescence images revealed a considerable increase in γ-H2AX adducts (pink dots) following TMZ treatment in cancer cells only, further amplified in the TMZ + CPZ combination (Fig. 6A). Notably, the effect of TMZ in RPE-1 cells was markedly reduced, resulting in an almost undetectable induction of strand breaks. In these non-cancer cells, only the CPZ + TMZ combo led to a detectable increase in DNA damage.

Quantitative cytofluorimetric analysis of phosphorylated histone H2AX yielded comparable results (Fig. 6B).

#### B. RPPA analysis reveals a CPZ-induced modulation of DNA damage pathway determinants

**Fig. 7:**
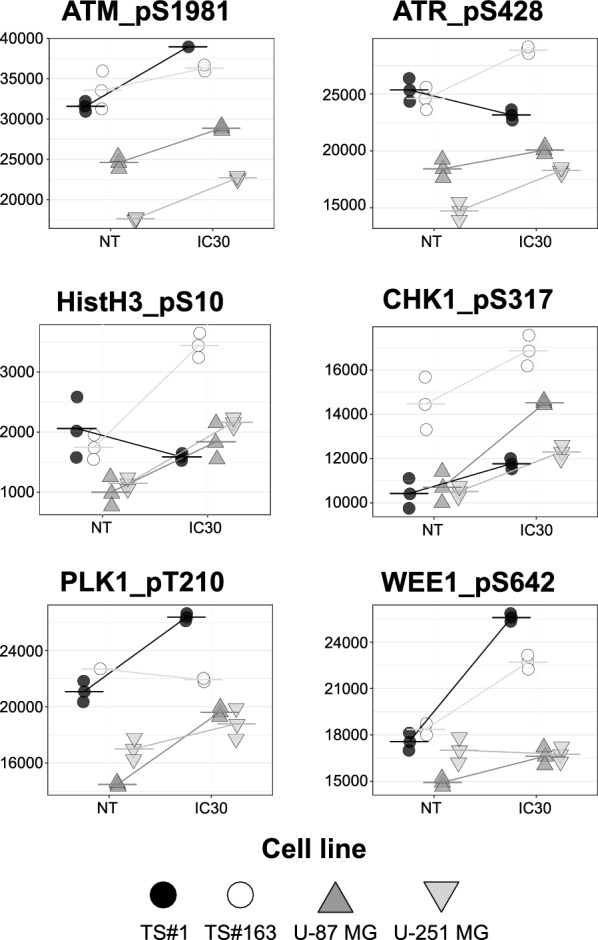
Interference of CPZ with DNA damage pathway determinants. The panels include selected plots of normalized RPPA levels (Arbitrary Units, AU) for endpoints implicated in DNA damage and DNA damage response pathways, as measured over dose response of CPZ (Control and IC30, from left to right) at 8 h. N = 3

In our previous work [33], we exploited the RPPA platform to investigate the pathway-level effects of CPZ on GBM cells. Employing a similar approach, we selected key endpoints involved in DNA damage sensing (ATM, ATR) and cell cycle markers (Histone H3) and checkpoint (CHK1, PLK1, WEE1) and measured their phosphorylated forms in GBM cells either left untreated or challenged with CPZ at IC30 for 8 h. Interestingly, most CPZ-treated cell lines showed an active DNA damage response, characterized by increased phosphorylation levels of both ATM and ATR as well as CHK1 [50, 52]. Notably, treatment with CPZ induced an overall increase of phospho-Histone H3 (Ser10), a bona fide marker of the onset of mitosis. Consistent with the presence of an induced mitotic signal, we observed that CPZ elevated the levels of phosphorylated, and thus inhibited, WEE1 along with increased active (i.e., phosphorylated at Thr210) PLK1 (Fig. 7). Collectively, these alterations suggest that the action of CPZ encompasses the unlocking of the cell cycle progression in the presence of damaged DNA, thus leading GBM cells towards mitotic catastrophe [31].

#### C. CPZ decreases DDR (by downregulation of Cx43)

**Fig. 8:**
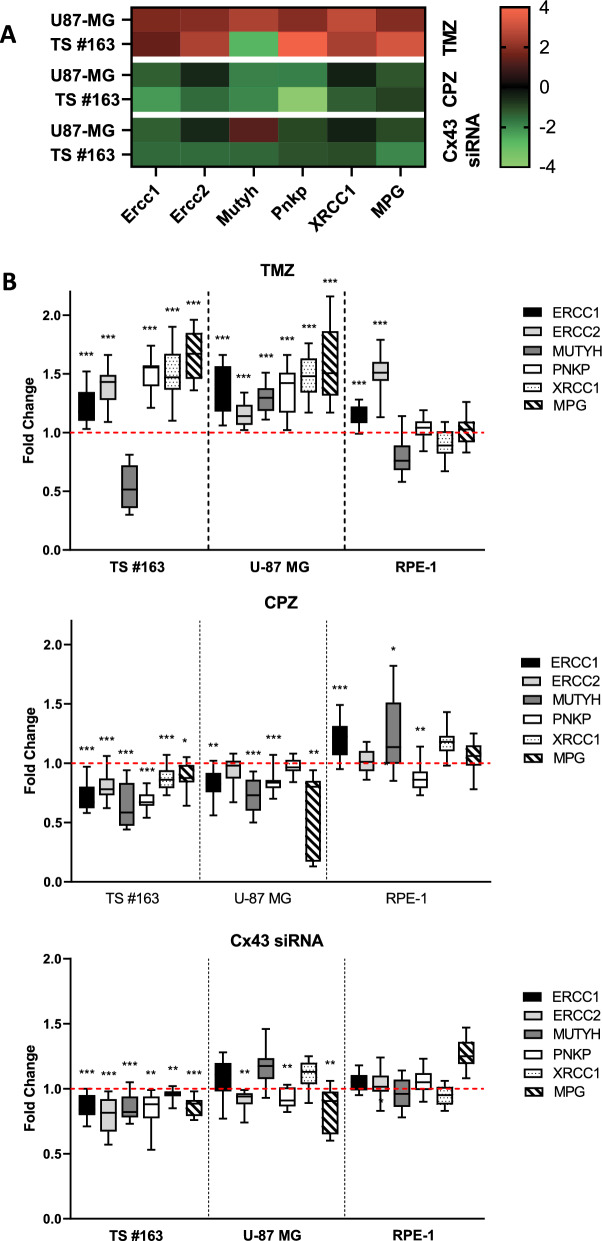
CPZ decreases DNA damage response (DDR) by means of Cx43 downregulation. **A** Heatmap showing Z-scores for relative DNA repair capacity. Repair capabilities above the respective controls are shown in red, while those below the controls in green. Z-units scale is on the right. TMZ treatment caused an increase in DNA repair genes expression; at the opposite, CPZ-treated and Cx43-silenced GBM cells highlighted a decrease in DNA repair capabilities, suggesting the involvement of Cx43 in hampering DNA damage repair. **B** Expression of a set of six DNA repair genes involved in glioma resistance to TMZ has been analyzed via RT-PCR analysis. Treated cell lines were exposed to TMZ for 6 days and CPZ or siCx43 for 48 h. While TMZ treatment induced a remarkable increase in DDR, CPZ-treated GBM cell lines, as well as Cx43-silenced GBM cell lines, exhibited a significant reduction in this set of DNA repair genes. RPE-1 non-cancer cell line differently responded to the treatments. Fold changes from three experiments are represented as boxes, with median, whiskers going from the smallest to the largest value and statistical significance vs untreated cells, referred as 1.0 (*p < 0.05; **p < 0.01; ***p < 0.001)

To assess the impact of drug treatment on DNA repair capacity in GBM cells, we analyzed, using RT-PCR, the expression of a set of genes involved in TMZ resistance in glioma cells [53]. TMZ treatment significantly increases DDR, mainly in GBM cells, thus contributing to chemoresistance, while CPZ-treatment led to a substantial decrease in the expression of these genes in GBM cell lines. Furthermore, Cx43 silencing in GBM cells resulted in a partial reduction in DDR, especially in neurospheres. Interestingly, RPE-1 non-cancer cells, treated with TMZ, CPZ, or Cx43-silenced, exhibited a different pattern of DDR gene expression, showing a milder modulation when compared with GBM cells. These results are summarized in Fig. 8, with panel A depicting a heatmap representing the expression trends for U-87 MG and TS#163 cells, and panel B showing box plots for U-87 MG, TS#163, and RPE-1 cells.

Overall, these data suggest that GBM cells and RPE-1 non-cancer cells employ distinct mechanisms to cope with DNA damage.

## Discussion

GBM, a highly aggressive brain tumor, continues to pose a significant therapeutic challenge with limited treatment options that have remained largely unchanged since 2005. In this context, drug repositioning has emerged as a promising approach, offering the potential to rapidly leverage existing clinically approved drugs with established safety profiles for GBM treatment, particularly in combination with TMZ. We have proposed the utilization of the antipsychotic CPZ in GBM therapy and recently completed a Phase II clinical trial to assess its efficacy and safety [37]. Alongside this clinical investigation funded on the known capability of this drug to interfere with the function of the post-synaptic monoamine receptors, additional past and ongoing experimental work has been addressed to unravel the intricate pharmacodynamics of this drug.

Building upon our previous work [31], we further delved into the oxidant properties of CPZ and investigated its potential to selectively hinder GBM aggressiveness by triggering the collapse of an antioxidant system already overwhelmed by the massive metabolic demands that characterize this tumor [54].

Indeed, in this study, we present compelling evidence that CPZ induces a clear pro-oxidative imbalance in the redox equilibrium of GBM cells. To summarize, this drug triggers the production of free radicals, NFR2 synthesis, increased levels of glutathione, activation of the ARE pathway and DNA damage, while simultaneously reducing DDR via Cx43 downregulation.

Combining TMZ with pro-oxidant drugs like CPZ could exploit a unique vulnerability in GBM cells. These cells have elevated ROS production and a compromised redox balance due to their high metabolic demands. This vulnerability originates from their compensatory upregulation of antioxidant systems, leading to increased NRF2 and GSH levels and subsequent activation of the ARE pathway. Classical chemo/radiotherapies or metabolic inhibitors induce ROS accumulation while simultaneously downregulate detoxifying enzymes, ultimately driving cancer cells to apoptosis. However, our studies show that CPZ-treated GBM cells do not undergo apoptosis [36], suggesting that alternative mechanisms are responsible for their demise. One possibility is that CPZ disrupts the neuroprotective function of Cx43 due to an increased oxidative stress exceeding the cellular tolerance threshold. This disruption could lead to unrepaired DNA damage and ultimately GBM cell death.

RPPA plots revealed a CPZ-induced protein phosphorylation pattern indicative of DNA damage and/or DDR imbalance. These DDR sensors play a critical role in cell cycle checkpoints [55–59], and their post-translational modifications suppress DNA repair mechanisms, forcing GBM cells carrying damaged DNA to prematurely enter mitosis, ultimately leading to cell death by mitotic catastrophe, as demonstrated in our previous study [31].

Connexins (Cxs), a family of transmembrane proteins involved in intercellular communication, play a crucial role in cellular protection against various stressors, including oxidative stress. Research has shown that enhanced cellular resistance to injuries mediated by Cxs, independent of gap junctions assembly, contributes to cell survival [60]. Notably, Cx43, the predominant component of astrocytic gap junctions, shields glioma rat cells from tamoxifen and UV irradiation [60] and human glioma cells from TMZ-induced damage, thereby conferring chemoresistance [15, 16]. In our study, we observed a significant reduction in Cx43 expression in GBM cells following CPZ exposure, which is compatible with a potential role for this drug in circumventing TMZ resistance.

TMZ is an alkylating agent that inflicts DNA damage, prompting treated GBM cells to activate their DNA repair mechanisms for survival. When these DNA repair systems falter, tumor cells become vulnerable to TMZ cytotoxic effects. Recent studies have shed light on DDR mechanisms [61], revealing that DNA repair capacity can predict TMZ resistance in GBM cells [62]. Additionally, the use of DNA repair inhibitors potentiates tumor cells sensitivity to radiation and TMZ treatment [63]. In this context, GSCs, equipped with enhanced DNA repair capabilities, play a pivotal role in TMZ resistance and GBM patient survival [64]. Boccard et al. identified a signature of DNA repair genes implicated in glioma resistance to TMZ and demonstrated that their downregulation significantly sensitizes GBM cells to chemotherapy [53]. Our experimental analysis of this DNA repair gene set revealed that: (a) treating GBM cells with TMZ elevates DNA repair genes expression; (b) treating GBM cells with CPZ diminishes DNA repair genes expression; (c) silencing Cx43 in GBM cells leads to a decrease in DNA repair gene expression, particularly in GSCs, the most treatment-resistant GBM cells. Building upon the synergistic effect of CPZ and TMZ in restraining GBM cell proliferation [36], we propose that CPZ could play a key role in overcoming TMZ resistance by reducing GBM cells’ DNA repair ability, potentially through Cx43 downregulation.

While these in vitro findings using diverse cell lines, including anchorage-dependent and stem-like neurospheres, are promising, further validation in vivo is crucial. However, traditional xenograft models in immunodeficient mice, while informative, carry intrinsic limitations. These animal models lack a functional immune system, which plays a critical role in tumor evolution and response to therapy [65, 66]. This limitation restricts their ability to fully mimic human GBM biology. In this context, the recently completed Phase II RACTAC clinical trial, in which the addition of CPZ to the standard adjuvant treatment with TMZ was assayed [37] provides a proof-of-concept demonstration of the effect of this combination in vivo directly assesses the effects in human patients, thus by far more exhaustive than an animal model.

**Fig. 9:**
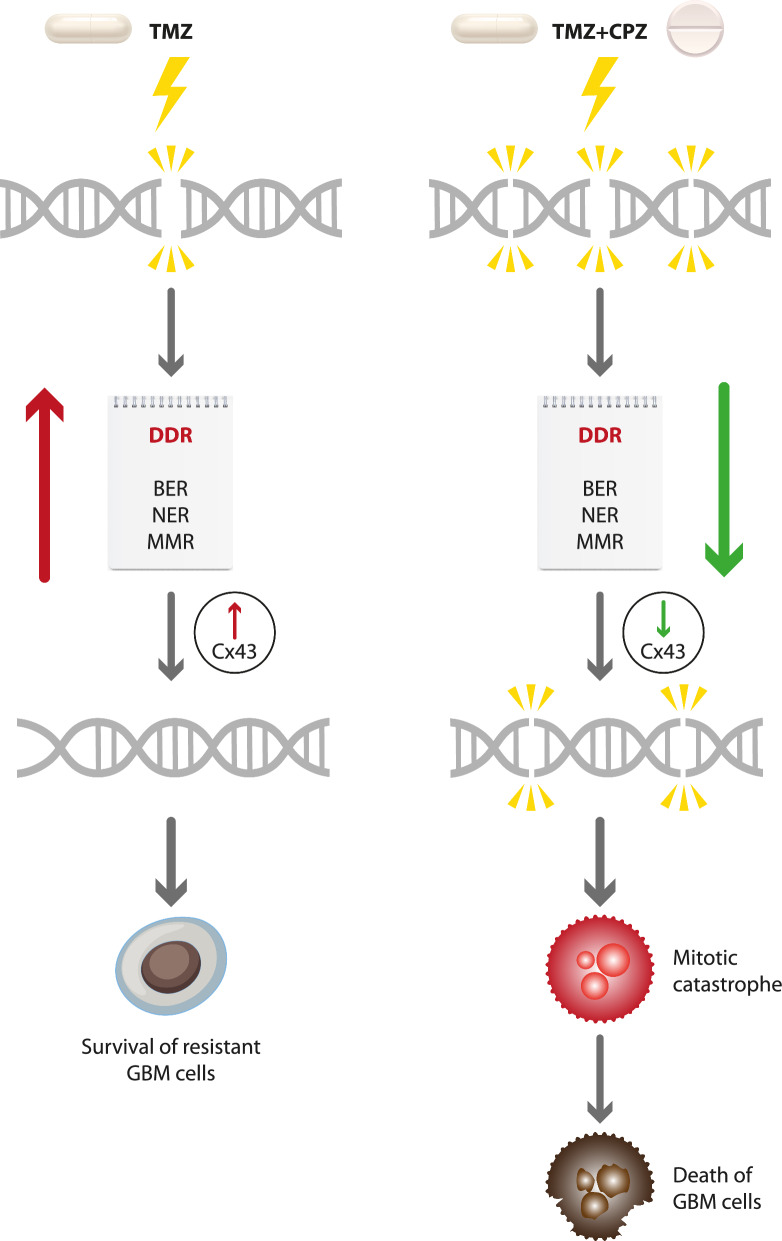
Schematic summary of the putative role of CPZ in overriding TMZ resistance in GBM cells. Results of the current study suggest that adding CPZ to TMZ treatment induces a decrease of DDR, as well as a reduction of Cx43 expression, pushing GBM cells with DNA damage to mitosis, finally resulting in mitotic catastrophe and subsequent cell death

As illustrated in our schematic diagram (Fig. 9), TMZ treatment triggers an escalation in DDR and upregulation of Cx43 expression, thus our model suggests that combining CPZ and TMZ treatments could induce a decrease in DDR and Cx43 expression, prompting an anticipated cell cycle progression, which ultimately culminates in mitotic catastrophe and cell death for GBM cells. Notably, silencing Cx43 appears to mimic the effects of CPZ treatment, as both conditions result in comparable patterns of reduced expression of DNA repair genes.

## Conclusions

Our study sheds new light on the multifaceted mechanisms underlying CPZ’s anti-cancer activity, particularly its ability to overcome TMZ resistance in GBM cells. Our in vitro studies provide compelling evidence that CPZ, when combined with first-line GBM therapy, amplifies DNA damage while concurrently downregulating a panel of DDR genes in GBM cells. Additionally, CPZ induces a relevant decrease in the neuroprotective protein Cx43. These combined effects steer DNA-damaged GBM cells toward cell death through mitotic catastrophe. These findings highlight the potential of CPZ as a repositionable drug, particularly for GBM patients struggling with TMZ resistance. Notably, our mechanistic insights corroborate the clinical efficacy of our recently completed phase II clinical trial [37], which integrates CPZ into the adjuvant phase of the standard first-line treatment regimen for newly diagnosed GBM patients with an unmethylated *MGMT* gene promoter.

### Supplementary Information

Supplementary Material 1.

Supplementary Material 2.

Supplementary Material 3.

Supplementary Material 4.

## Data Availability

Raw data are available at the following link: https://gbox.garr.it/garrbox/s/FNuP7HlR4VPhI70

## Abbreviations

GBM: Glioblastoma
TMZ: Temozolomide
BER: Base excision repair
MGMT: Methylguanine-DNA methyltransferase
MMR: Mismatch repair
GSC: Glioma stem cells
CX-43 or GJA1: Connexin-43 or gap junction A1
CPZ: Chlorpromazine
TM: Tumor microtubes
DRD2: Dopamine receptor D2
ROS: Reactive oxygen species
DCFH_2_-DA: 2′,7′-Dichlorodihydrofluorescein diacetate
RPPA: Reverse-phase protein microArrays
NRF2: Nuclear factor erythroid 2-related factor 2
GSH: Glutathione or reduced glutathione
GSSG: Oxidized glutathione
ARE: Antioxidant response element
HO-1: Hemoxygenase 1
NQO1: NAD(P)H dehydrogenase quinone 1
MnSOD: Manganese superoxide dismutase
γ-H2AX: Gamma variant of histone H2AX
DDR: DNA damage response
ATM: Ataxia-telangiectasia mutated protein
ATR: Ataxia telangiectasia and Rad3-related protein
CHK1: Checkpoint kinase 1
PLK1: Polo like kinase 1
WEE1: G2 checkpoint kinase
GAPDH: Glyceraldehyde-3-phosphate dehydrogenase
ERCC1: Excision repair 1
ERCC2: Excision repair 2
MUTYH: MutY DNA glycosylase
PNKP: Polynucleotide kinase
XRCC1: X-ray repair cross complementing 1
MPG: N-methylpurine DNA glycosylase
