# Supplementary material for: Chlorpromazine overcomes temozolomide resistance in glioblastoma by inhibiting Cx43 and essential DNA repair pathways
Source: J Transl Med. 2024 Jul 18;22:667. doi: 10.1186/s12967-024-05501-3 (PMC11256652; doi:10.1186/s12967-024-05501-3)
Supplement: Supplementary file 3 — Supplementary Material 3. [file 12967_2024_5501_MOESM3_ESM.pdf]

Figure S1

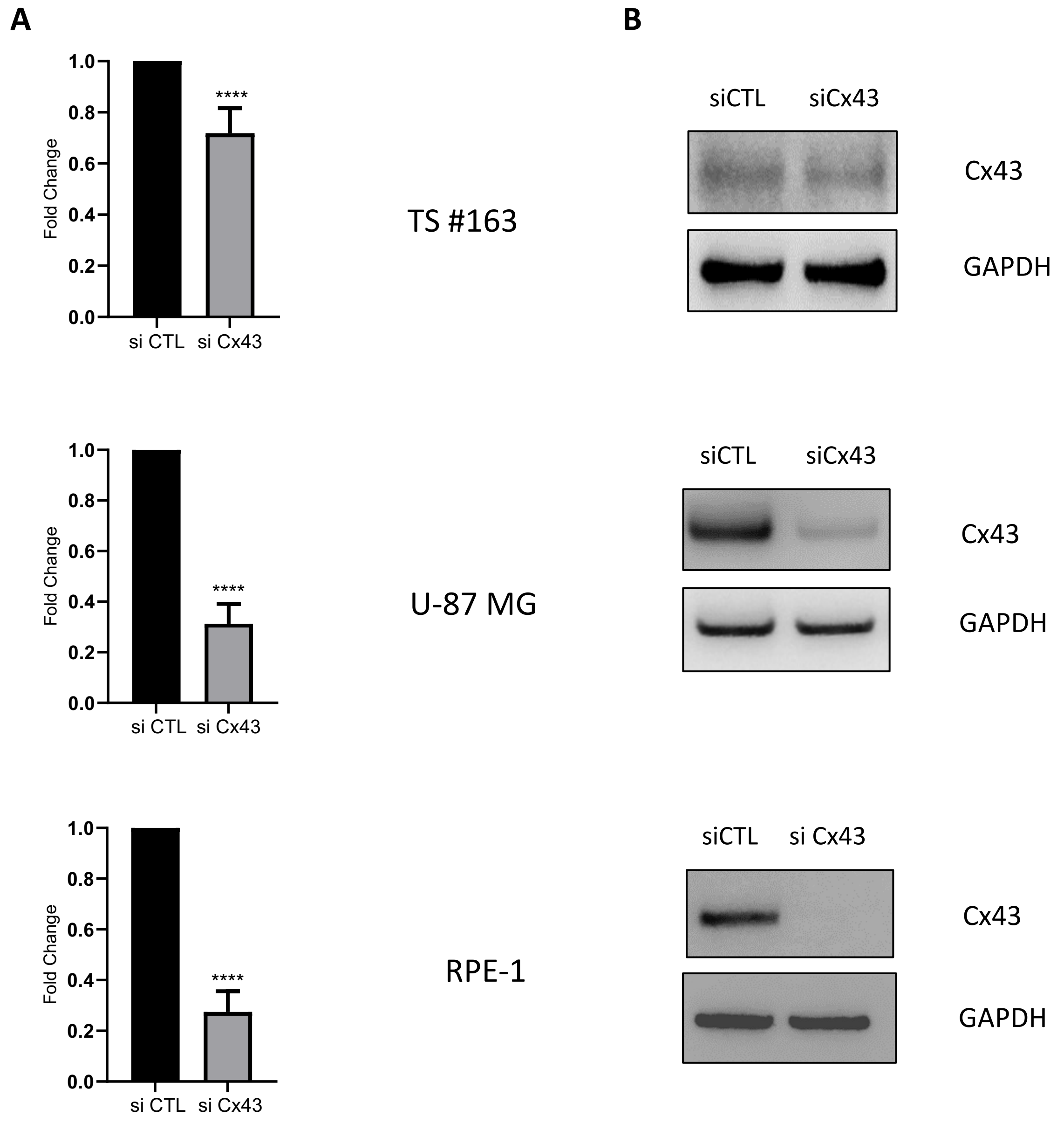

Cx43 downregulation by siRNA transfection. After transient transfection of neurospheres (TS#163), GBM cells (U-87 MG) and non tumoral cells (RPE-1), CX43 silencing was evaluated both at transcriptional level, by means RT-PCR (A), and at protein level, by means western blotting analyses (B).
