# Supplementary material for: Chlorpromazine overcomes temozolomide resistance in glioblastoma by inhibiting Cx43 and essential DNA repair pathways
Source: J Transl Med. 2024 Jul 18;22:667. doi: 10.1186/s12967-024-05501-3 (PMC11256652; doi:10.1186/s12967-024-05501-3)
Supplement: Supplementary file 4 — Supplementary Material 4. [file 12967_2024_5501_MOESM4_ESM.pdf]

Figure S2

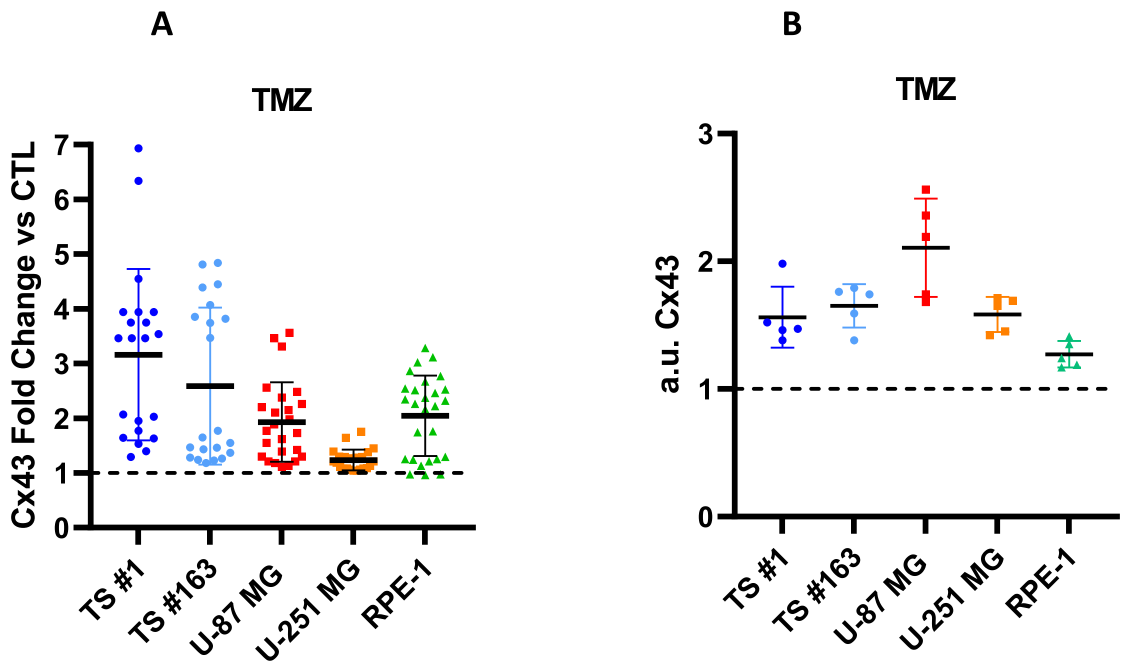

TMZ increases Cx43 expression in all cell lines. Anchorage-dependent U-87 MG and U-251 MG GBM cells and RPE-1 non-cancer cells, as well as GBM-patients derived neurospheres, were exposed to TMZ or solvent (CTL) for six days. Subsequently, CX43 expression was analyzed both at transcriptional level, by means RT-PCR (**A**), and at protein level, by means cytofluorimetric analyses (**B**) showing a notable increase of Cx43 expression in all cell lines relative to each control (referred as 1.0).
